# From indigenous screening to pilot fermentation: comprehensive characterization of *Bacillus subtilis* YZ01 as a novel food-grade probiotic candidate

**DOI:** 10.3389/fmicb.2025.1624208

**Published:** 2025-08-29

**Authors:** Xiaoyu Cao, Yu Zhang, Qianqian Xu, Hai Yan

**Affiliations:** School of Chemistry and Biological Engineering, University of Science and Technology Beijing, Beijing, China

**Keywords:** *Bacillus subtilis*, probiotics, safety assessment, pilot-scale fermentation, biodegradation

## Abstract

*Bacillus subtilis* YZ01, a novel strain isolated from the feces of a healthy individual, demonstrated potent *in vitro* biodegradation of uric acid (54.13%) and nucleosides (41.21%). Phylogenomic analysis of 2,198 single-copy orthologous genes confirmed strain YZ01’s taxonomic identity, while genome mining uncovered five circular antimicrobial peptide clusters. *B. subtilis* has been extensively utilized in the food industry for decades and is on the U.S. Food and Drug Administration’s GRAS (Generally Recognized as Safe) list. Integrated genomic and phenotypic characterization revealed the probiotic properties and safety profile of *B. subtilis* YZ01, and proposed pathways for its biodegradation of uric acid and nucleosides. *In vitro* assays indicated that *B. subtilis* YZ01 exhibits excellent acid and bile salt tolerance as well as good adhesion, supported by the presence of stress-response genes, adhesion-associated surface proteins, and bile salt hydrolase genes. Antibiotic resistance genes and virulence factors were identified and confirmed to be safe through associated tests (antibiotic resistance, hemolytic assay, cytotoxicity assay and biogenic amine production). Furthermore, pilot-scale fermentation in a 100 L fermenter achieved high-density biomass production (6.12 × 10^10^ CFU/mL) under optimized culture conditions, underscoring its industrial scalability. These findings highlight *B. subtilis* YZ01’s bioactive properties, food-safe profile, industrial feasibility, and promising potential as a probiotic candidate for the development of functional foods aimed at improving hyperuricemia.

## 1 Introduction

Probiotics, defined as live microorganisms that confer health benefits to the host when administered in adequate amounts (FAO/WHO), have gained significant attention for their functional potential (Hill et al., 2014). Among probiotic varieties, *Bacillus* species, notably spore-forming strains like *Bacillus subtilis*, have been widely utilized as food-grade probiotics for decades, due to its versatility and safety (Lee et al., 2019). In the food industry, several strains of *B. subtilis* are also approved for direct use in food products, such as *B. subtilis* natto in fermented soy products (Schallmey et al., 2004), *B. subtilis* DE111^®^ in functional food (Mazhar et al., 2023), and clinically validated probiotics including *B. subtilis* CU1 (Lefevre et al., 2017) and *B. subtilis* R0179 (Tompkins et al., 2008). These strains enhance nutrition and gut health, while their antimicrobial compounds (e.g., subtilin) act as natural preservatives to inhibit pathogens and extend shelf life.

However, despite the widespread use of *B. subtilis*, the development of novel *B. subtilis* probiotics faces critical knowledge gaps that must be addressed. Strain-specific safety validation remains imperative due to genomic variations that necessitate rigorous assessment of virulence factors and antibiotic resistance profiles (Brutscher et al., 2022). Furthermore, few isolates have undergone characterized for industrial scalability through high-density fermentation feasibility studies. Critically, developing probiotics with independent intellectual property (IP) is essential for translational applications. To this end, targeted screening of indigenous microbial resources enables the discovery of strains with both functional novelty and commercial viability. Recent advances highlight the unique advantages of *B. subtilis* over conventional probiotics like lactic acid bacteria (LAB), including exceptional gastric acid resistance (Lee et al., 2019), extended shelf-life via sporulation, and enzymatic uric acid biodegradation through conserved uricase pathways (Gong et al., 2018). *In vitro* studies have demonstrated the ability of *B. subtilis* strains to degrade uric acid and purines (Rouf and Lomprey, 1968; Pustake et al., 2019), while its application in fermenting medicinal herbs for the management of hyperuricemia has also been investigated (Wang et al., 2023).

Here, we report the isolation and characterization of *B. subtilis* YZ01, a newly isolated strain from the feces of a healthy individual through proprietary screening protocols designed to secure IP rights. This strain exhibited significant biodegradation capacity of uric acid and its precursors, aligning with its genomic arsenal of purine-metabolizing enzymes. Crucially, our study transcends biodegradation evaluation to deliver a comprehensive probiotic dossier. We integrated whole-genome sequencing, *in silico* safety profiling, and phenotypic validation to systematically evaluate strain YZ01’s probiotic potential and demonstrated its industrial viability through high-density fermentation, bridging the gap between laboratory discovery and commercial application.

## 2 Materials and methods

### 2.1 Bacterial strains and culture conditions

*B. subtilis* YZ01 was isolated and screened from the feces of a healthy individual, which purchased from Beijing Fumate Biotechnology Co., Ltd. (Beijing, China). Single colonies of strain YZ01 were grown in Luria-Bertani (LB) broth at 37 °C, 200 rpm for 24 h. *Staphylococcus aureus* was cultured in LB broth under the same conditions as above.

### 2.2 Whole genome sequencing and genome annotation

Genomic DNA of *B. subtilis* YZ01 was extracted using MagPure Bacterial DNA Kit (D6361-02, Magen, Shanghai, China), and then its integrity, concentration, and purity were determined. The whole genome was sequenced on the Illumina NovaSeq 6,000 platform at Sangon Biotech (Shanghai, China). Genome assembly was done using SPAdes v3.15 and the Gapfiller v1.11 was used for filling gaps. Gene prediction was generated using the Prokka v1.10, and gene functional annotation was mainly based on COG (Clusters of Orthologous Groups of proteins), GO (Gene Ontology), KEGG (Kyoto Encyclopedia of Genes and Genomes) and CAZy (Carbohydrate-active Enzymes) database. The predicted genes were compared with the CARD (Comprehensive Antibiotic Research Database) and VFDB (Virulence Factors of Pathogenic Bacteria) for identifying antibiotic resistance genes and virulence factors.

### 2.3 Pangenome and comparative analysis

The assembled genome was compared with the NT (Nucleotide sequence) database using NCBI Blast+. Based on the matching scores, homologous strains were identified. Pangenome analysis and core-genome alignment were conducted on seven *B. subtilis* strain (Supplementary Table S1) genomes obtained from the RefSeq database with the *B. subtilis* YZ01 genome. This analysis was performed using the Roary plugin^1^ (Wu et al., 2022).

### 2.4 *In vitro* uric acid and nucleosides biodegradation by whole cell

To evaluate the *in vitro* biodegradation ability of uric acid and nucleosides (both inosine and guanosine), 2 mL of the 24-h-old *B. subtilis* YZ01 culture solution (OD_600_ = 8.23) was centrifugated (8,000 × g, 10 min, Thermo Fisher Scientific, Osterode am Harz, Germany) and washed twice with stroke-physiological saline solution, then resuspended in 750 μL phosphate buffer solution (PBS, 0.1 M, pH 7.4) containing uric acid (1.68 g/L) or nucleosides (1.12 g/L), respectively. After 24-h reaction, equal volume of 0.5 M NaOH was added to terminate the biodegrading reaction and the mixture was filtered by a 0.22-μm membrane filter for further quantification by high-performance liquid chromatography (HPLC, Shimadzu LC-20AT, Tokyo, Japan). Standard curves correlating concentration (0.02–0.10 g/L) with peak area were established for uric acid, inosine, and guanosine, with all regression equations yielding *R*^2^ > 0.999. Blank controls (without *B. subtilis* YZ01 cells) were included in all biodegradation assays under identical experimental conditions.

The biodegradation ratio was calculated: (C0 – Ct)/C0 × 100%, where C0: initial concentration of uric acid or nucleosides (g/L), Ct: residual concentration of uric acid or nucleosides in the sample (g/L).

### 2.5 Determination of uric acid biodegradation capacity by crude enzyme

Crude enzyme activity assays were performed using cell lysates of *B. subtilis* YZ01. Cultures (24-h-old) were harvested by centrifugation (8,000 × g, 10 min, 4 °C), washed twice with sterile physiological saline to remove residual medium, and resuspended in 5 mL PBS. Cells were lysed through ultrasonication (360 W, 25 min total duration, 10 s pulse-on/5 s pulse-off cycles) in an ice-water bath. The lysate was centrifuged (8,000 × g, 10 min, 4 °C), and the supernatant (cell-free extract) was collected. Uric acid was added to the extract at a final concentration of 0.50 g/L, followed by incubation at 37 °C for 12 h. Protein concentration in the cell-free extract was determined using a BCA assay kit (Beyotime, Shanghai, China).

### 2.6 Probiotic properties

#### 2.6.1 Identification genes encoding bacteriocins and secondary metabolite prediction

The potential bacteriocin-encoding gene clusters were identified in the genome using BAGEL4 v.1.2. Moreover, secondary metabolic gene cluster prediction was conducted using antiSMASH v7.1.0.

#### 2.6.2 Acid and bile salt tolerance

To evaluate acid and bile salt tolerance, 16-h-old *B. subtilis* YZ01 cultures (Supplementary Figure S1) were prepared at an approximate concentration of 10^8^ CFU/mL in LB broth. The bacterial suspension was then transferred into acidic conditions (pH 2.0, 2.5, 3.0, 4.0, 5.0) and LB broth containing bile salt (0.1, 0.2, 0.3, 1, and 2% (w/v)), followed by incubation at 37 °C for 3 h and 5 h, respectively (Wu et al., 2022). The samples were collected and plated on LB agar to determine the number of surviving bacteria compared to the control group. Survival rates were calculated as (%) = (Final (log CFU/mL)/Initial (log CFU/mL)) × 100.

#### 2.6.3 Auto-aggregation and cell surface hydrophobicity assays

Auto-aggregation and cell surface hydrophobicity assays were performed as described by Ait Seddik et al. (2017). Briefly, 24-h-old *B. subtilis* YZ01 cells were re-suspended in PBS at 10^8^ CFU/mL after centrifugation (8,000 × g, 15 min). Subsequently, cell suspensions were mixed by vortexing and incubated 37 °C for 6 h. At the same time, cell suspensions were mixed with xylene, chloroform and ethyl acetate (v: v = 3: 1) by vortexing for 3 min, and then incubated at room temperature until phase separation. Absorbance of liquid phase or lower aqueous phase of the above suspension was detected by a spectrophotometer (INNESA, Shanghai, China) at 600 nm. Auto-aggregation percentage or cell surface hydrophobicity was calculated as (%) = 1−(A1/A0) × 100, where A1 represented the final absorbance or aqueous phase after phase separation, A0 showed the initial absorbance.

### 2.7 Safety assessment

#### 2.7.1 Hemolytic activity

Hemolytic assay of *B. subtilis* YZ01 was carried out using Columbia blood agar (Babio, China) as described by previous research (Wang et al., 2024). *S. aureus* was used as positive control for hemolytic activity.

#### 2.7.2 Antibiotic susceptibilities

According to the guidelines of Clinical and Laboratory Standards Institute (CLSI), the antibiotic susceptibilities of *B. subtilis* YZ01 were determined by the macrodilution broth method against eight common antibiotics (Brutscher et al., 2022), including vancomycin, ampicillin, tetracycline, gentamicin, chloramphenicol, clindamycin, rifampicin, and ciprofloxacin (Solarbio, Beijing, China). The minimum inhibitory concentration (MIC) values were determined by visual observation of the turbidity, and the results were expressed as sensitive or resistant according to CLSI M45 for *Bacillus* strains.

#### 2.7.3 Amino acid decarboxylase test

Refer to previous study methods (Wang et al., 2024), *B. subtilis* YZ01 bacterial suspension (100 μL) were inoculated into micro-biochemical identification tube containing different amino acids (lysine, arginine, and ornithine) (Hopebio, Qingdao, China) and amino acid decarboxylase control, respectively. And then, 300 μL of sterilized liquid paraffin was added into each tube and incubated at 37 °C for 24 h. The color changes in the broth were observed.

#### 2.7.4 Cytotoxicity assay

The cytotoxicity of *B. subtilis* YZ01 to Caco-2 cells was determined according to previous research method (Fu et al., 2022). Caco-2 cells were grown in Minimum Essential Medium (MEM) supplemented with 20% (v/v) fetal bovine serum at 37 °C and 5% CO_2_ to the logarithmic growth phase. Cells were detached using Trypsin (0.25%) and then made the cell suspension. Caco-2 cells were seeded into 96-well plates (20,000/well) and cultured in MEM for 24 h. After centrifugation (8,000 × g, 10 min), the cells of *B. subtilis* YZ01 were resuspended in MEM, while the supernatant was filtered through a 0.22-μm membrane filter to obtain cell-free supernatant. After that, MEM of the 96-well plate was replaced with *B. subtilis* YZ01 cells suspension (10^8^ CFU/mL) or cell-free supernatant, and maintained for another 24 h. Add 10 μL of CCK-8 reagent to each well containing 100 μL of cell culture medium and incubated for another 4 h. The same volume of the MEM was added to the blank control wells. Absorbance was measured at 450 nm, and the percentage of cell viability was calculated as cell viability (%) = 100 × (A/Ab), where A is the absorbance of the treated wells and Ab is the absorbance of the blank control wells.

### 2.8 Culture optimization and pilot-scale fermentation

This study was based on modified LB broth and explored the optimal culture conditions (Yang et al., 2023) for *B. subtilis* YZ01 by changing its temperature (20, 30, 37 and 40 °C), initial pH (6, 7, 8, 9), inoculum size (0.1, 0.5, 1 and 2%), carbon source (glycerinum, glucose, saccharose and sodium lactate), nitrogen source (yeast power, peptone, urea and NH_4_Cl), and carbon-to-nitrogen (C/N) ratio (5:1, 10:1, 20:1 and 30:1). The growth was characterized using OD_600_ values. After that, a pilot-scale fermentation of *B. subtilis* YZ01 using a 100-L bioreactor (Bailun, Shanghai, China) was conducted under the optimized culture conditions. The C/N ratio is calculated as:


$$C/N=\frac{\left(0.4\times G)+(0.45\times P\right)}{0.14\times P}$$

Where *G* represents the mass of glucose (g), *P* represents the mass of peptone (g).

### 2.9 Statistical analysis

All results were reported as the mean and standard deviation (SD) from three independent experiments. The statistical analysis of the data was conducted by SPSS 22.0 and Origin 2021.

## 3 Results and discussion

### 3.1 Overview of the *Bacillus subtilis* YZ01 genomic characteristics

**Figure 1:**
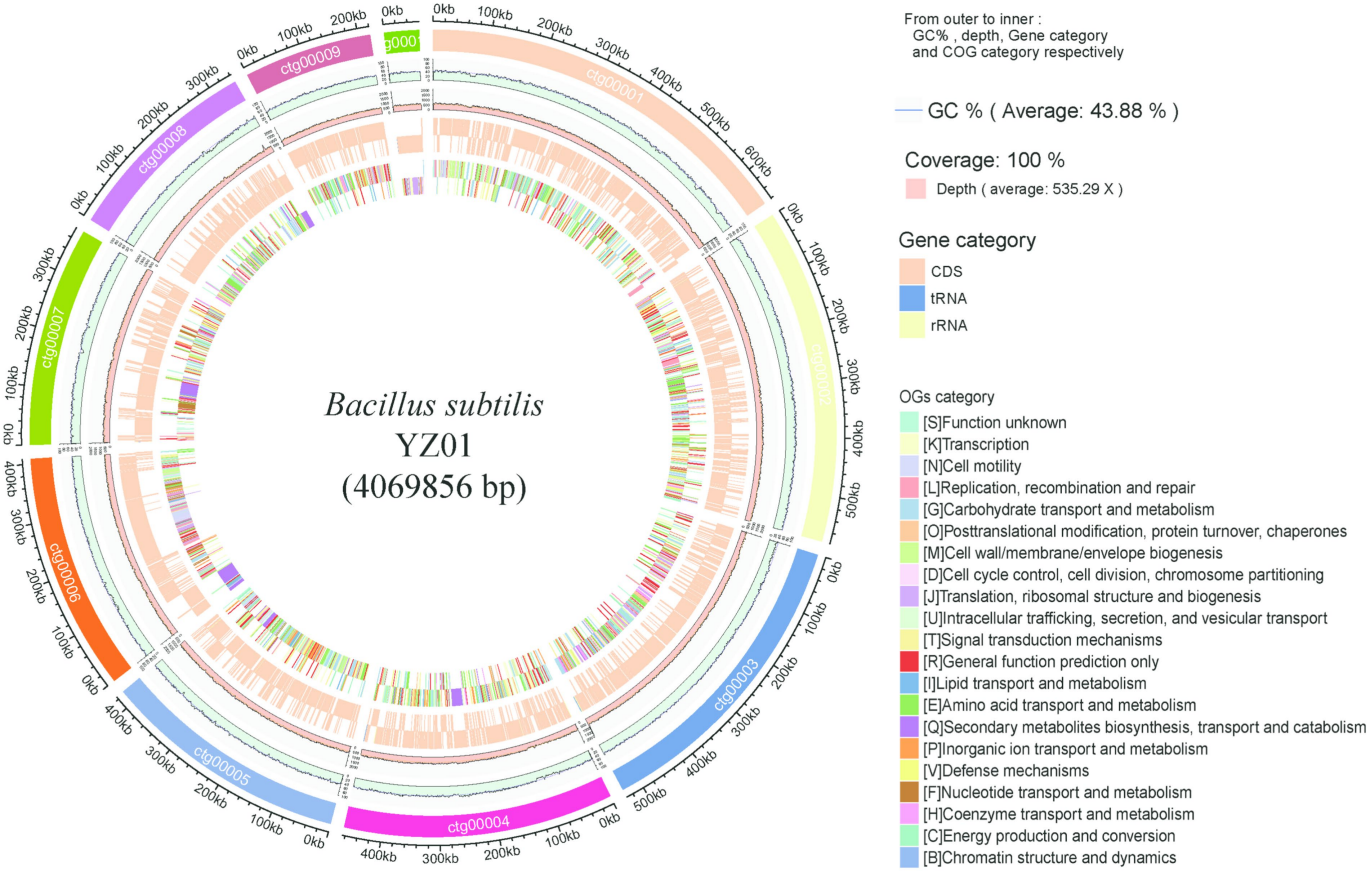
Genome visualization and annotation of *B. subtilis* YZ01. The ring provides details from outer to inner: GC%, depth, gene category and COG category.

The draft genome sequence of *B. subtilis* YZ01 contained 19 contigs of 4,069,856 bp with an average GC content of 43.88% (Figure 1), which were consistent with the values reported by *B. subtilis* strains (Anderson et al., 2005). This genome predicted a total of 4,261 genes, containing 4,177 protein-coding genes, 9 rRNA genes, 74 tRNA genes, 1 ncRNA genes. The raw genome sequence of *B. subtilis* YZ01 has been deposited in the SRA of the NCBI under the accession number PRJNA1209663.

### 3.2 Gene annotation of *Bacillus subtilis* YZ01

**Figure 2:**
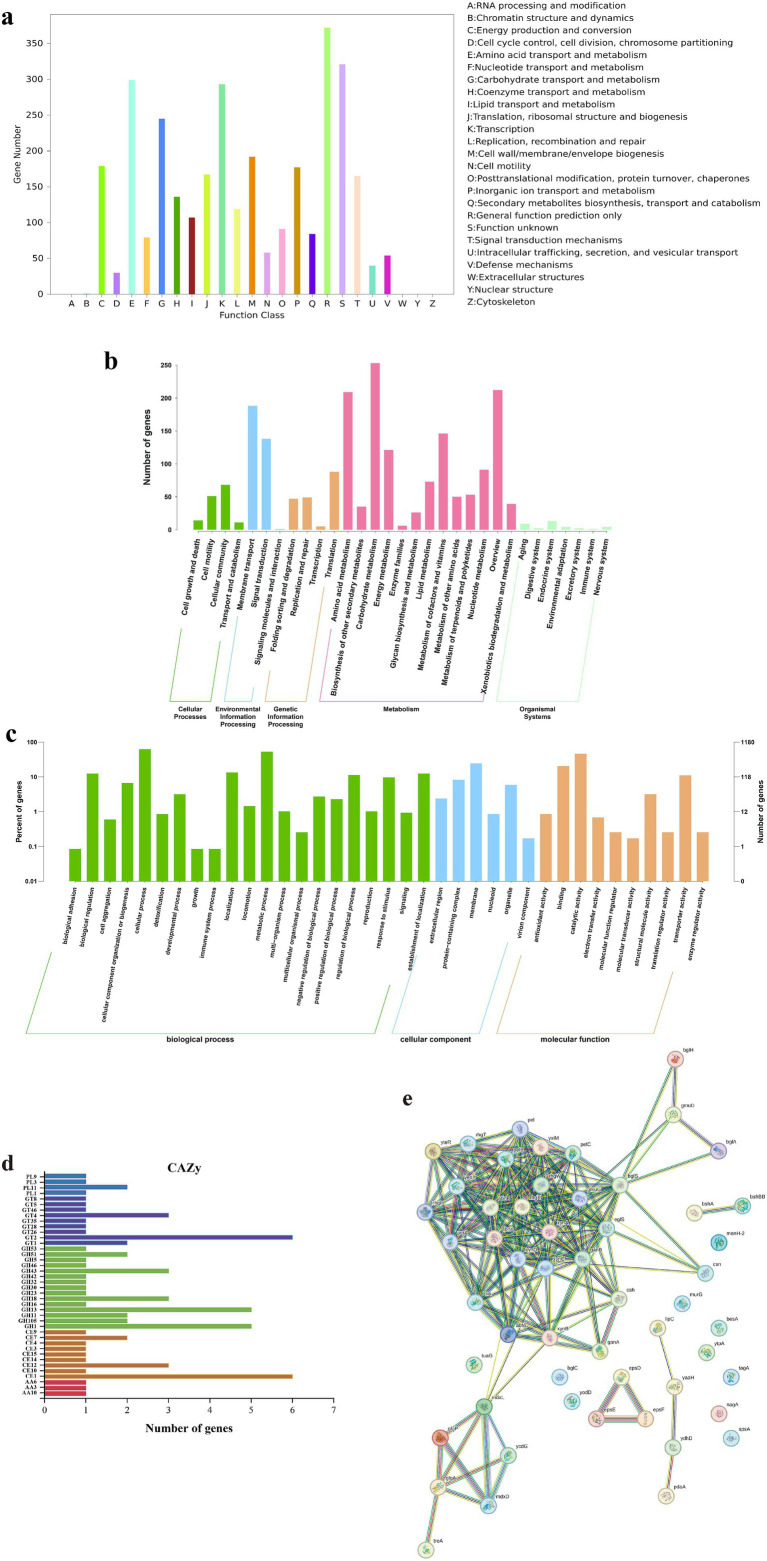
Gene functional annotation of *B. subtilis* YZ01. COG database annotation **(a)**. GO database annotation **(b)**. KEGG database annotation **(c)**. CAZy database annotation **(d)**. Protein–protein interactions of CAZy **(e)**.

Functional annotation of *B. subtilis* YZ01 genome reveled extensive metabolic capabilities (Figure 2). COG analysis classified 2,907 genes into 25 functional categories (Figure 2a). Dominant categories included Amino acid transport and metabolism (E, 299 genes), Carbohydrate transport and metabolism (G, 245 genes), Lipid transport and metabolism (I, 109 genes), Secondary metabolites biosynthesis, transport and catabolism (Q, 84 genes), and Nucleotide transport and metabolism (F, 79 genes). This profile reflects robust metabolic diversity, supporting rapid proliferation, stress tolerance, and potential purine metabolism modulation (Wang et al., 2024). GO annotation assigned 1,180 genes to three functional domains (Figure 2b): Biological Process, Cellular Component, and Molecular Function. These results reflect the relationship between genes and corresponding functions. Within the Biological Process domain, cellular processes predominated, followed by metabolic processes. Among Cellular Components, membrane-associated terms were most frequent. For Molecular Function, catalytic activity constituted the largest group. KEGG pathway analysis identified 1,314 metabolism-associated genes (Figure 2c). Top subcategories were Carbohydrate metabolism (253 genes), Amino acid metabolism (209 genes), Metabolism of cofactors and vitamins (146 genes), Energy metabolism (121 genes), and Nucleotide metabolism (91 genes). CAZy annotation detected 72 carbohydrate-active enzymes (Figure 2d), primarily Glycoside Hydrolases (GH, 30 genes), Glycosyl Transferases (GT, 17 genes), and Carbohydrate Esterases (CE, 17 genes), with fewer Polysaccharide Lyases (PL, 5 genes) and Auxiliary Activities (AA, 3 genes). These enzymes facilitate carbohydrate degradation and biosynthesis, critical for biodegradation and nutrient cycling (Diale et al., 2021).

Protein–protein interaction (PPI) analysis of CAZy-annotated enzymes was performed using STRING v.12.0 (confidence score ≥0.7; Figure 2e). Due to the absence of strain YZ01 in the database, interactions were inferred from *B. subtilis* 168 homologs. Key PPIs, such as oligosaccharide transporters (*mdxL*/*mdxD*) coupled with glycogen metabolism enzymes (*glgA*/*glgP*), enable synergistic polysaccharide degradation, enhancing metabolic adaptability in complex herbal substrates. This provides a theoretical basis for the subsequent development of functional foods to improve hyperuricemia by utilizing *B. subtilis* YZ01 fermentation with food and medicine homology (Wang et al., 2023).

Notably, the PPI network highlighted a membrane-associated exopolysaccharide (EPS) export complex (*epsD-epsE-epsF*). EPS production may directly reduce serum uric acid by inhibiting xanthine oxidase activity and promoting renal excretion via urate transporter modulation (e.g., ABCG2) (Ullah et al., 2025). Concurrently, EPS exerts prebiotic effects that reshape gut microbiota composition, suppress purine-metabolizing pathobionts, and attenuate systemic inflammation, collectively mitigating hyperuricemia pathogenesis.

### 3.3 Evolutionary position

**Figure 3:**
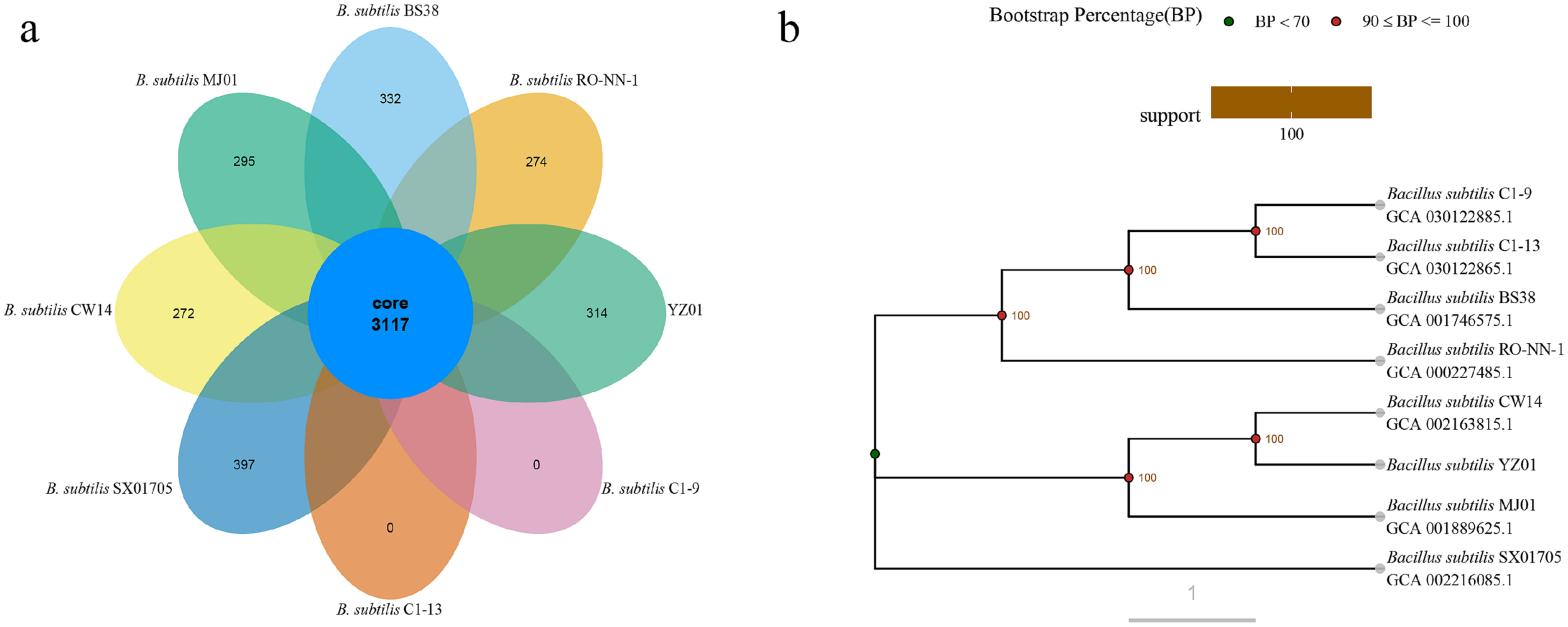
Venn diagram of gene families **(a)** and evolutionary tree **(b)**. Orthologs in eight different *B. subtilis* strain gene family Venn graph. A phylogenetic tree based on core single copy orthologs genes demonstrated the close genetic distance of all eight *B. subtilis* strains genomes.

Phylogenomic analysis provides critical insights into *B. subtilis* strain diversity and taxonomic classification (Wu et al., 2022). Gene families serve as robust markers for reconstructing evolutionary trajectories and identifying functional divergence (Thornton and DeSalle, 2000), while functional conservation enables predictive annotation of uncharacterized proteins (Rodríguez del Río et al., 2023). As shown in Supplementary Figures S2a,c, pangenome analysis of eight *B. subtilis* strains revealed a core genome comprising 3,117 genes (47.5%), along with 1,563 dispensable genes (23.8%) and 1884 specific genes (28.7%). Among these, 2,430 genes were classified into 21 COG categories (Supplementary Figure S2b). As shown in Figure 3a, eight ellipses represented the eight *B. subtilis* genomes, and the data on each region represented the number of gene families that only appeared in the species. Genes were found only in each strain form *B. subtilis* BS38, RO-NN-1, C1-9, C1-13, SX01705, CW14, MJ01 are 332, 274, 0, 0, 397, 272 and 295, respectively. The phylogenetic tree was constructed based on core single copy orthologs genes using the Neighbor-joining clustering method and elucidated the closest relationship observed between *B. subtilis* YZ01 and *B. subtilis* CW14 (4,282,516 bp) or *B. subtilis* MJ01 (4,108,329 bp) (Figure 3b). The genome size of *B. subtilis* YZ01 was similar to those of two strains, but it was 212.66 kb and 38.473 kb smaller than that of CW14 and MJ01, respectively.

### 3.4 Biodegradation of uric acid and nucleosides by *Bacillus subtilis* YZ01

**Figure 4:**
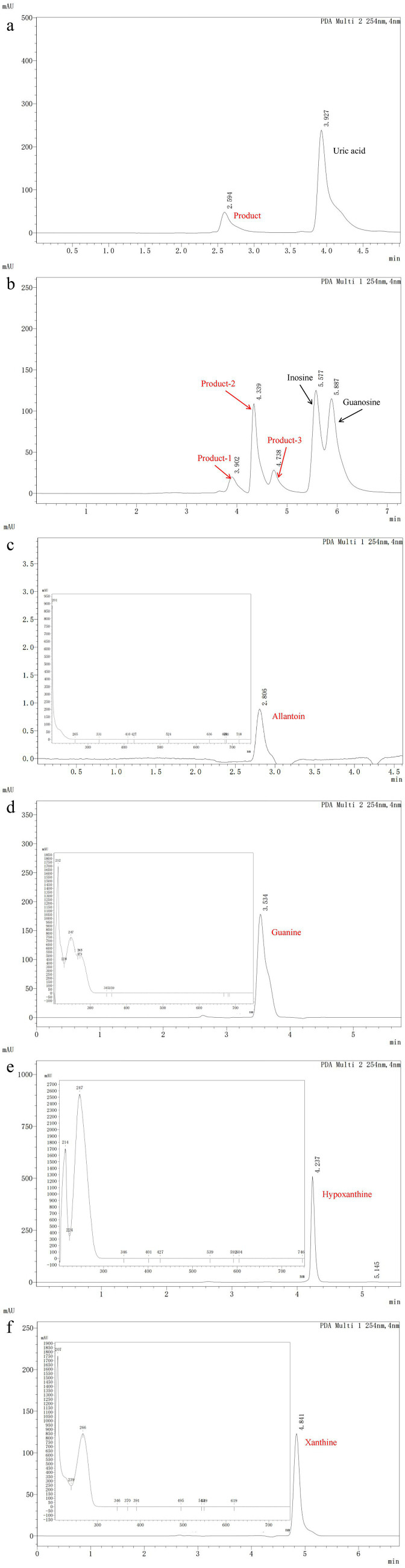
Detection of uric acid **(a)** and nucleosides **(b)** biodegradation products within 24 h by *B. subtilis* YZ01. Detection peaks of allantoin **(c)**, guanine **(d)**, hypoxanthine **(e)**, and xanthine **(f)** standards.

To determine the uric acid and nucleosides biodegrading ability of *B. subtilis* YZ01, we added the bacterium to PBS solutions containing uric acid or nucleosides for cultivation. Results showed that *B. subtilis* YZ01 biodegraded 41.21 ± 2.83% of nucleosides and 54.13 ± 1.97% of uric acid within 24 h. Meanwhile, crude enzyme containing a protein concentration of 0.43 ± 0.08 g/L, could remove 0.41 ± 0.04 g/L of uric acid within 12 h (Supplementary Figure S3). Although the uric acid degradation capacity of *B. subtilis* YZ01 (54.13% reduction from 1.68 g/L in 24 h) was lower than the engineered *Saccharomyces cerevisiae* strain expressing *Vibrio vulnificus* uricase (0.0614 ± 0.0035 g/h/OD) (Wang et al., 2025), it significantly exceeded that of the uricolytic *Limosilactobacillus fermentum* JL-3 (40.90% reduction from 1.68 g/L in 24 h) (Wu et al., 2021). HPLC results revealed absorption peaks for uric acid, inosine and guanosine at 3.927, 5.577 and 5.887 min (Figures 4a,b). Additionally, peaks corresponding to their biodegradation products were observed at 2.594, 3.902, 4.339, and 5.887 min. Based on previous studies (Kuo et al., 2021; Wu et al., 2021; Li et al., 2023), we hypothesized that uric acid was biodegraded to allantoin, while inosine and guanosine were biodegraded to guanine, hypoxanthine, and xanthine. Subsequently, we determined the retention times and spectral profiles of these reference standards (Figures 4c–f). The matching chromatographic peaks and spectral patterns between the biodegradation products and the standards confirmed their identity.

Genomic analysis of *B. subtilis* YZ01 identified genes and enzymes associated with the biodegradation of uric acid and nucleosides (Table 1). The general metabolic pathway of uric acid biodegradation proceeds via three steps: it is first converted to 5-hydroxyisourate, then decomposed into 2-oxo-4hydroxy-4-carboxy-5-ureidoimidazoline, and eventually forms allantoin spontaneously (Wu et al., 2021). This process is attributed to *B. subtilis* YZ01 expressing the *pucL* gene (encoding a uricase homolog) and the *pucM* gene (encoding 5-hydroxyisourate hydrolase) (Zhao et al., 2022). For the biodegradation of inosine and guanosine, these compounds are first converted to guanine and hypoxanthine via the action of purine-nucleoside phosphorylase. Subsequently, guanine and hypoxanthine are further transformed into xanthine by guanine deaminase and xanthine dehydrogenase, respectively (Maiuolo et al., 2016). These findings, supported by genomic sequencing analysis and HPLC results, provide strong evidence for elucidating the pathways of uric acid, inosine, and guanosine biodegradation in *B. subtilis* YZ01.

**Table 1:** Uric acid and nucleosides biodegrading-related genes in *B. subtilis* YZ01 genome.

Gene ID	Gene name	Product	Enzyme commission
ctg00005-02610	*pucM*	5-hydroxyisourate hydrolase	EC 3.5.2.17
ctg00005-02611	*pucL*	Uric acid degradation bifunctional protein PucL	EC 1.7.3.3
ctg00007-03367	*hpxO*	FAD-dependent urate hydroxylase	EC 1.14.13.113
ctg00009-03900	*punA*	Purine-nucleoside phosphorylase	EC 2.4.2.1
ctg00005-02601, ctg00005-02603, ctg00005-02604, ctg00005-02606	*pucA* *pucC* *pucD* *pucE*	Putative xanthine dehydrogenase	EC 1.17.1.4
ctg00002-01132	*guaD*	Guanine deaminase	EC 3.5.4.3

### 3.5 Probiotic properties

#### 3.5.1 Genes encoding bacteriocins and secondary metabolite

**Figure 5:**
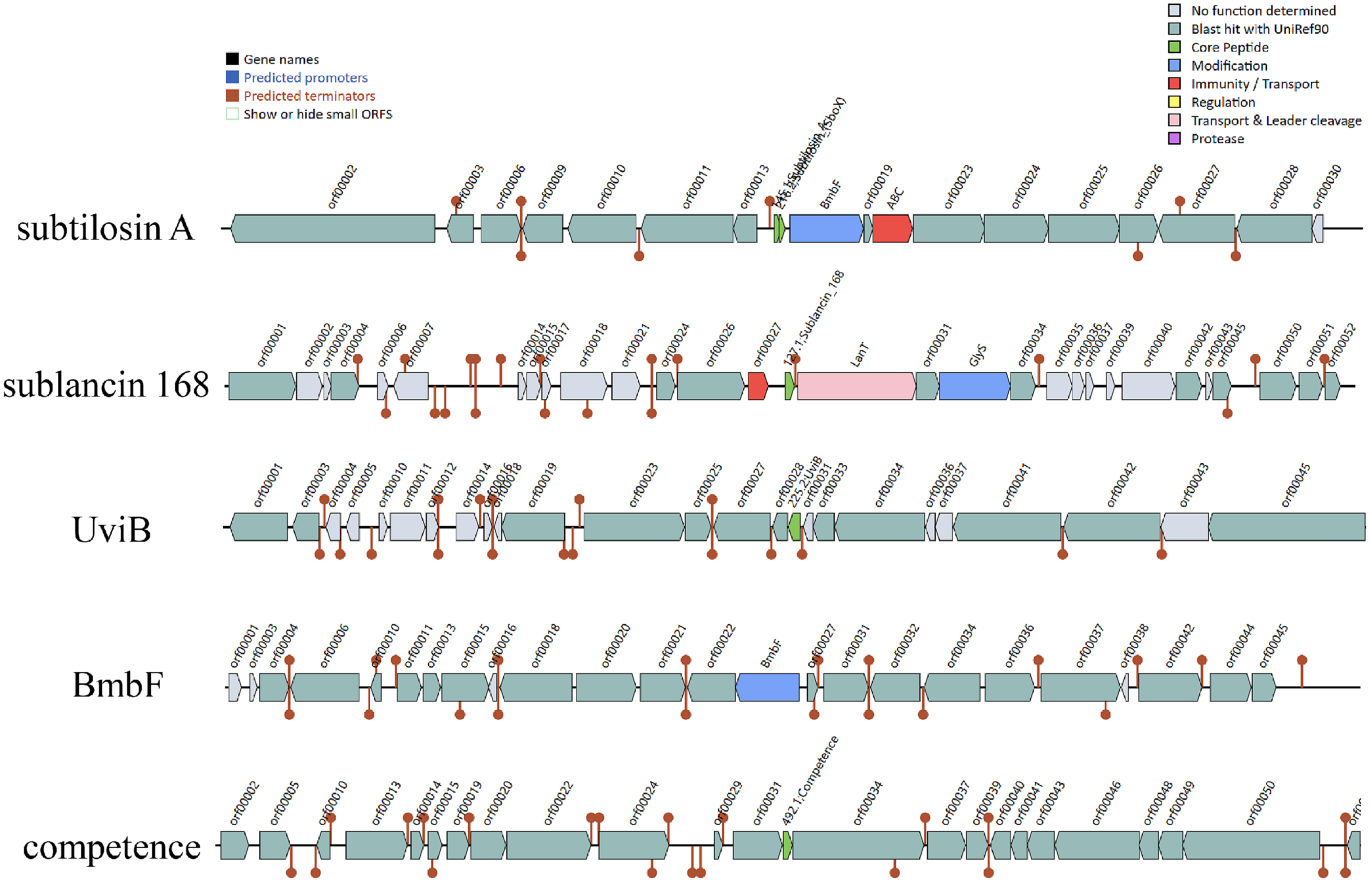
The gene cluster for antimicrobial peptides were predicted with online servers BAGEL4. Five contigs encoding genes potentially related to the biosynthesis of subtilosin A, sublancin, UviB, BmbF and competence were identified in *B. subtilis* YZ01 genome.

The *Bacillus* genus is known to produce diverse antimicrobial peptides, including post-translationally modified peptides (RiPPs), non-ribosomal peptides (NRPs), and polyketides (PKs) (Abriouel et al., 2011). Antimicrobial activity is an important probiotic property that enables strains to combat gastrointestinal infections. This activity enhances environmental adaptability and colonization capacity, ensuring sustained expression of health-promoting functions such as immunomodulation and metabolic support (Mu et al., 2024). AntiSMASH and BAGEL4 were used to identify putative genes in *B. subtilis* YZ01 genome involved in antimicrobial compounds. AntiSMASH identified fourteen gene clusters associated with the biosynthesis of terpene, NRPS, T3PKS, and RiPP-like compounds. *In silico* analysis with BAGEL4 identified five putative gene clusters associated with the biosynthesis of subtilosin A, sublancin 168, UviB, BmbF and competence bacteriocin (Figure 5). The subtilosin A cluster contains genes encoding precursor peptides and post-translational modification enzymes, with the mature bacteriocin being extracellularly secreted via an ABC transporter (Kawai et al., 2004). The UviB gene cluster encodes a prophage-derived holin-like perforin that inhibits pathogenic bacteria such as *Listeria monocytogenes* in dairy environments (Cloeckaert et al., 2019). The competence bacteriocin gene cluster comprises regulatory genes (*comQ*, *comX*, *comP*, *comA*) that orchestrate the quorum sensing system in *B. subtilis* for interbacterial communication (Schneider et al., 2002). Additionally, the sactipeptides cluster features the *BmbF* gene, encoding a key enzyme that mediates thioether bond formation in precursor peptides during RiPPs biosynthesis, with subtilosin A being generated through this modification mechanism (Flühe et al., 2012).

#### 3.5.2 Analysis of stress-related genes in *Bacillus subtilis* YZ01

As a probiotic strain, *B. subtilis* YZ01 exhibits robust genomic adaptations to environmental stresses and safety considerations critical for industrial and consumer applications. Genomic analysis identified key genes underpinning its stress resilience (Supplementary Table S2), including those encoding catalases and superoxide dismutase, which scavenge reactive oxygen species to mitigate oxidative damage, and sporulation-related genes that enable survival under harsh conditions via dormant spore formation. The strain’s stress-responsive capacity is further regulated by the *rsbV*, *rsbW*, and *rsbX* genes, which modulate sigma factor activity to orchestrate balanced stressosome-mediated responses during environmental challenges (Khadka et al., 2025). According to Guidelines for the Evaluation of Probiotics in Food (FAO/WHO), bile salt hydrolase (BSH) activity is a primary *in vitro* assessment criterion for probiotics. Genomic analysis identified five *bsh* genes in *B. subtilis* YZ01. Additionally, antiporters maintain intracellular pH stability by expelling excess protons, thereby preventing protein structural integrity (Söylemez-Milli et al., 2025). These integrated genomic traits, encompassing oxidative resistance, pH homeostasis, stress signaling, and protective metabolite synthesis, collectively validate the environmental adaptability and safety profile of *B. subtilis* YZ01, supporting its potential application as a probiotic strain in food.

#### 3.5.3 Tolerance tests

**Figure 6:**
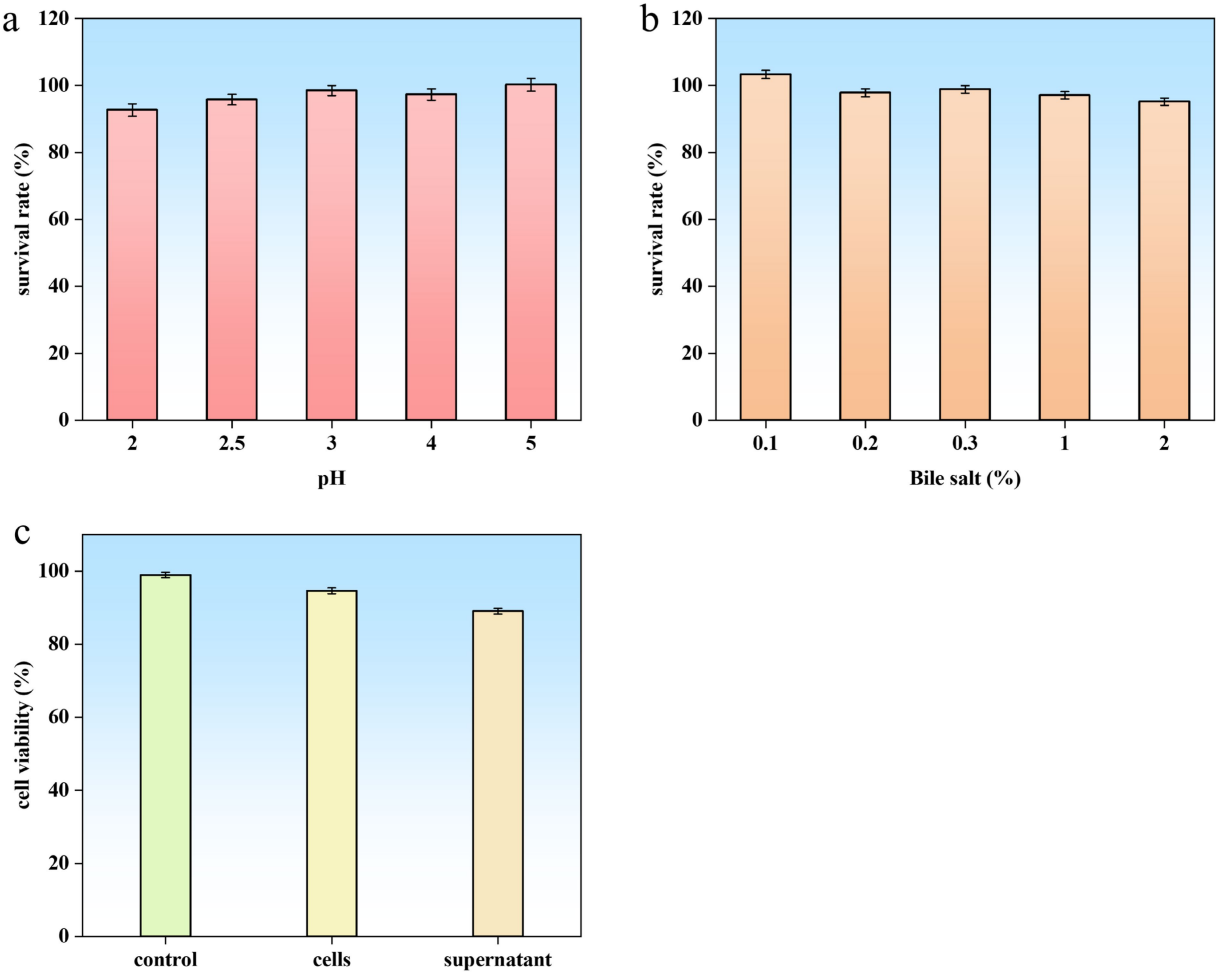
Probiotic properties and safety assessment of *B. subtilis* YZ01. Acid **(a)** and bile salt **(b)** tolerance of strain YZ01. Cytotoxic effect of strain YZ01 cells and supernatant on Caco-2 cells after 24 h of treatment **(c)**.

Exceptional tolerance to gastrointestinal stresses, including acidic gastric conditions and elevated bile salt concentrations, is a critical determinant of probiotic viability and functionality. *B. subtilis* YZ01 demonstrated remarkable resilience, maintaining survival rates >90% under both low-pH challenge and high bile salt exposure (Figures 6a,b). This performance aligns with the documented gastric (88.98%) and intestinal (98.60%) viability of the probiotic strain *B. subtilis* DC-11 (Chaudhari et al., 2024). Such robust acid-bile tolerance ensures sufficient viable cells reach the intestinal tract, fulfilling the prerequisite for intestinal colonization and subsequent exertion of beneficial effects. This adaptive capability not only enhances survival during gastrointestinal transit but also underscores its potential for functional food applications.

#### 3.5.4 Cell surface properties of *Bacillus subtilis* YZ01

Robust adhesion capabilities further validate the probiotic attributes of *B. subtilis* YZ01. Auto-aggregation assays demonstrated a time-dependent increase, rising from 35.68% at 2 h to 71.25% at 4 h. This strong self-aggregation propensity, indicative of potent intercellular interactions, facilitates biofilm formation and intestinal colonization. Notably, strain YZ01’s auto-aggregation capacity was significantly higher than that of *B. subtilis* DC-11 (18.33%) (Chaudhari et al., 2024) and *B. subtilis* P229 (49.18% at 4 h) (Jeon et al., 2018).

Surface hydrophobicity, a key indicator of host cell adhesion affinity, was assessed via solvent partitioning assays, yielding values of 19.02% (xylene), 37.24% (chloroform), and 35.94% (ethyl acetate). Genomic analysis identified key adhesion-related genes (e.g., *ctpB*, *luxS*, *pglF*, *groL*, *dnaK*) within *B. subtilis* YZ01 strain. Collectively, these findings demonstrate the strain’s pronounced ability to adhere to hydrophobic intestinal surfaces, a critical probiotic trait enabling competitive exclusion of pathogens, modulation of gut microbiota, and interaction with host immune cells. This adhesion-mediated colonization enhances both probiotic efficacy and sustained delivery of bioactive metabolites, supporting *B. subtilis* YZ01’s potential for modulating gut health.

### 3.6 Safety properties

Following GB 31615.2-2025 (National Food Safety Standard for the Assessment of Microbial Agents in Food, Evaluation Method b), which mandates comprehensive whole-genome sequencing analysis for virulence, antimicrobial resistance, and toxin production genes in food-use microbial strains, *B. subtilis* YZ01 was evaluated. Analysis identified 240 putative virulence factor genes in *B. subtilis* YZ01 genome (Supplementary materials). Among these, only four genes (*clpC*, *bslA*, *clpP*, and *capC*) exhibited >75% similarity to entries in the VFDB. Critically, these genes contribute to bacterial adaptability, persistence, and host interaction, functions essential for probiotic efficacy, such as pathogen competition, gastrointestinal stress resistance, and beneficial immune modulation (Lemos and Burne, 2002). Their presence does not confer functional virulence or toxicity in *B. subtilis* YZ01; instead, they mediate fundamental cellular processes and stress adaptation vital for probiotic function within the host.

Crucially, no genes encoding hemolysin or enterotoxin were detected. Phenotypically, *B. subtilis* YZ01 exhibited non-hemolytic activity on blood agar after 48 h at 37 °C, contrasting with the *β*-hemolysis displayed by *S. aureus* (Supplementary Figure S4a). This confirms safety regarding hemolysis for food applications. Cytotoxicity testing using Caco-2 cells revealed no significant reduction in cell viability after 24 h exposure to either *B. subtilis* YZ01 cell suspensions or culture supernatants, compared to controls (Figure 6c). Based on these genomic and phenotypic analyzes, *B. subtilis* YZ01 is preliminarily deemed safe. While *B. subtilis* has a well-established history of safe use, further *in vivo* studies, including acute oral toxicity and genotoxicity assessments as specified in GB 31615.2-2025, are recommended to comprehensively validate strain-specific safety and ensure regulatory compliance.

Comparison with CARD identified eleven putative antibiotic resistance genes associated with resistance to aminoglycosides (*ykkD*), tetracyclines (*ykkC*), lincosamides (*vmlR*, *lmrB*), chloramphenicol (*bmr*) and fluoroquinolones (*blt*). However, no corresponding phenotypic resistance was observed. *In vitro* susceptibility testing confirmed strain YZ01’s sensitivity to eight commonly used antibiotics (Table 2), aligning with probiotic safety requirements mandating the absence of transmissible antibiotic resistance.

**Table 2:** MIC values observed for *B. subtilis* YZ01 against tested antibiotics.

Antibiotics	MIC cut-off values (μg/mL)	MIC observed (μg/mL)	Interpretation
Vancomycin	4	0.5	S
Ampicillin	0.25	0.06	S
Tetracycline	4	0.06	S
Gentamicin	4	0.13	S
Chloramphenicol	8	8	S
Clindamycin	0.5	0.13	S
Rifampicin	1	0.06	S
Ciprofloxacin	1	0.25	S

This discrepancy may arise from low/absent gene expression, non-functional protein products due to post-translational modifications, or pseudogenization. Notably, the annotated streptomycin resistance gene aligns with studies indicating intrinsic streptomycin resistance is a conserved genomic feature within the *Bacillus* genus (Brutscher et al., 2022).

Biogenic amines (BAs) are nitrogenous organic compounds primarily produced via amino acid decarboxylation (Jeon et al., 2018). Common examples include cadaverine (lysine-derived), putrescine (ornithine-derived), and histamine (arginine-derived). In humans, BAs serve essential physiological functions as neurotransmitters and regulatory molecules, modulating cellular growth and metabolism (Wójcik et al., 2020). Certain BAs may also contribute to flavor and aroma development during food fermentation. Nevertheless, excessive BA accumulation poses significant health risks, including allergic reactions and toxicity (Doeun et al., 2017). Consequently, monitoring BA levels in fermented products (e.g., cheese, fish, wine) is essential for consumer safety. To assess the BA production potential of *B. subtilis* YZ01, we performed qualitative assays using decarboxylase broths supplemented with the precursor amino acids lysine, ornithine, and arginine. Results confirmed the absence of lysine decarboxylase, ornithine decarboxylase, and arginine decarboxylase activity (Supplementary Figure S4b).

### 3.7 Pilot-scale fermentation under optimal conditions

We systematically evaluated the influence of cultivation temperature, initial pH, inoculum size, carbon source, nitrogen source and C/N ratio on *B. subtilis* YZ01 growth (Supplementary Figure S5). Optimal growth conditions were identified as: temperature: 37 °C; initial pH: 7.0; inoculum size: 2% (v/v); carbon source: glucose; nitrogen source: peptone; C/N ratio: 5:1. Using these optimized parameters, pilot-scale fermentation was conducted in a 100-L bioreactor over 24 h. At harvest, the final OD_600_ of culture achieved 31.09 (viable cell count: 6.12 × 10^10^ CFU/mL). Following centrifugation, drying, and pulverization, 305.59 g of bacterial biomass powder was obtained. These results demonstrate the successful pilot-scale of *B. subtilis* YZ01 production, providing a robust experimental foundation for its future applications within the food industry.

## 4 Conclusion

This study characterizes the genomic and phenotypic profile of *B. subtilis* YZ01, proposing the pathways for uric acid and nucleosides biodegradation in this novel strain. Phylogenetic reconstruction using 1,352 single-copy orthologs revealed *B. subtilis* YZ01’s closest evolutionary affinity with *B. subtilis* CW14 and *B. subtilis* MJ01. Genomic screening confirmed absence of functional antibiotic resistance genes, virulence factors, and toxin-coding sequences, with safety further validated through hemolysis assays, cytotoxicity testing, antibiotic susceptibility profiling, and BAs production assessment. Genome annotation identified antimicrobial biosynthetic clusters, adhesion-associated genes, and stress-response mechanisms (extreme temperatures, acid, bile salt, and oxidative stress tolerance). Phenotypically, *B. subtilis* YZ01 exhibited robust gastrointestinal fluid resistance, high adhesion capacity, and industrial-scale fermentation viability. These findings collectively position *B. subtilis* YZ01 as a probiotic candidate for functional foods and dietary supplements development.

## Data Availability

The original contributions presented in the study are publicly available. This data can be found at: https://www.ncbi.nlm.nih.gov/sra, accession number PRJNA1209663.

## Footnotes

^1^ https://sanger-pathogens.github.io/Roary/
