# Supplementary material for: From indigenous screening to pilot fermentation: comprehensive characterization of Bacillus subtilis YZ01 as a novel food-grade probiotic candidate
Source: Front Microbiol. 2025 Aug 29;16:1624208. doi: 10.3389/fmicb.2025.1624208 (PMC12426190; doi:10.3389/fmicb.2025.1624208)
Supplement: Supplementary file 2 [file Supplementary_file_1.docx]

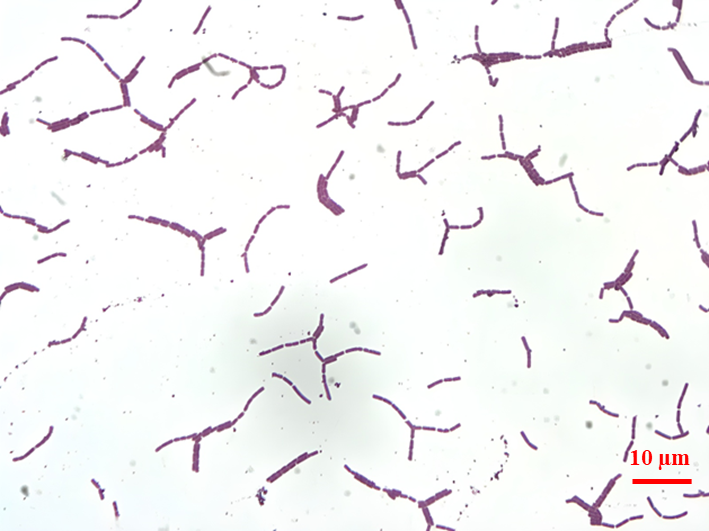


Figure. S1 Morphology (1000×) of 16-h-old *Bacillus subtilis* YZ01 under the microscope.

Figure. S2 Pie chart of homologous gene distribution (a). Cluster of Orthologous Groups (COG) classification of predicted proteins (b), the Y-axis represents the number of genes within each category, while the X-axis denotes the 21 functional COG categories. Pan-genome presented a core and accessory gene by comparing with the genomes of *B*. *subtilis* (c).


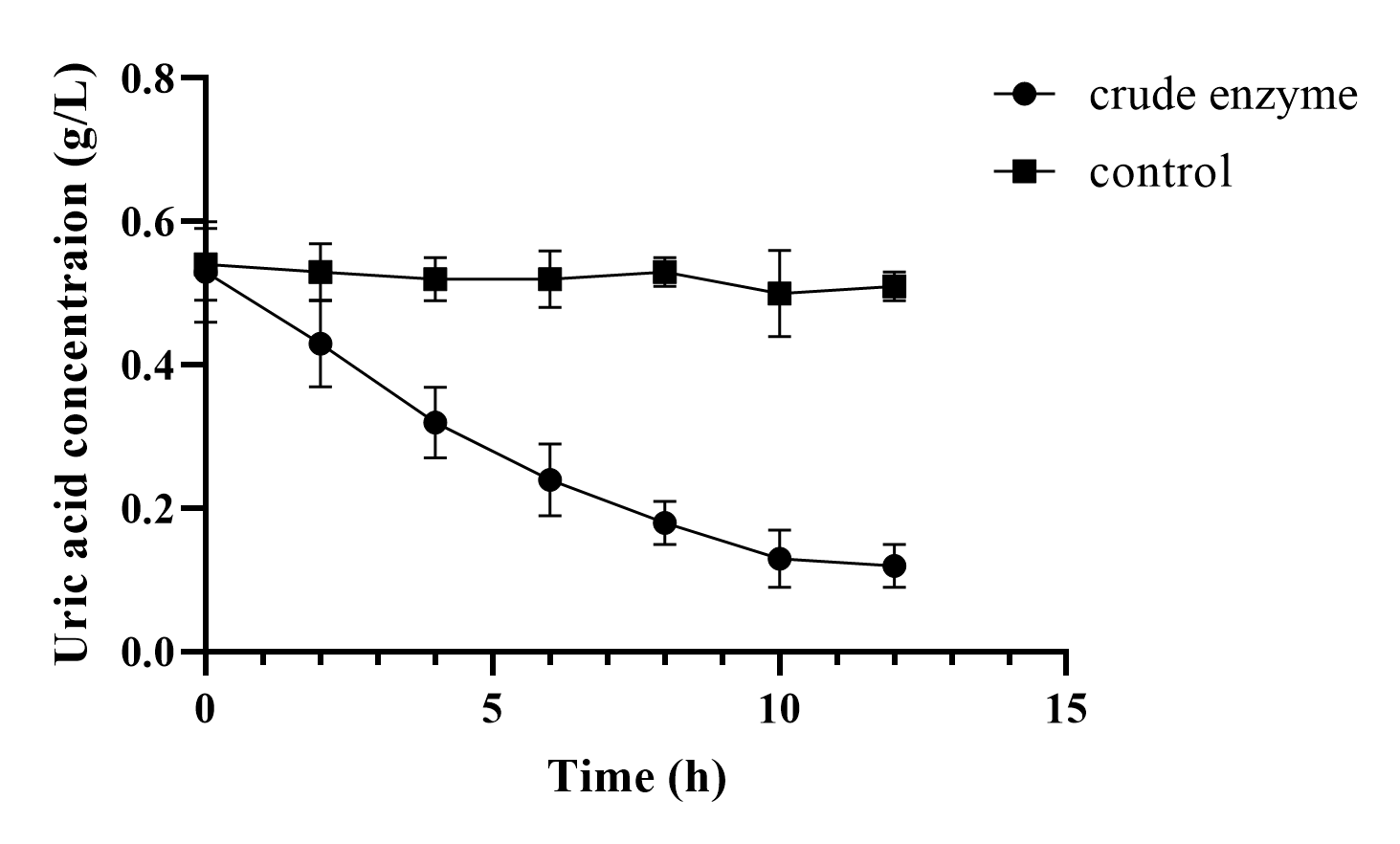


Figure. S3 Biodegradation of uric acid by crude enzyme (*B. subtilis* YZ01’s cell-free extract). Data are expressed as mean ± SD from three independent biological replicates (n=3). PBS containing uric acid without the crude enzyme was incubated under identical conditions for 12 h as the control.

Figure. S4 *In vitro* safety assessment of *B*. *subtilis* YZ01. Strain YZ01 showed non-hemolysis (a, left). The positive control *S*. *aureus* produced an obvious zone of complete-hemolysis (a, right). *B. subtilis* YZ01 exhibited no potential to produce biogenic amine (b).

Figure. S5 Effects of temperature (a), initial pH (b), inoculum size (c), carbon source (d), nitrogen source (e) and C/N (f) on growth of *B. subtilis* YZ01 cultured for 24 h.

Table S1 Genome of seven strains of the *B*. *subtilis* species.

| NCBI strain identifiers | Accession | Genome size (Mb) | CDS |
| --- | --- | --- | --- |
| *Bacillus subtilis* MJ01 | GCA 001889625.1 | 4.1 | 4204 |
| *Bacillus subtilis* BS38 | GCA 001746575.1 | 4.0 | 4250 |
| *Bacillus subtilis* RO-NN-1 | GCA 000227485.1 | 4.0 | 4130 |
| *Bacillus subtilis* C1-9 | GCA 030122885.1 | 4.1 | 4214 |
| *Bacillus subtilis* C1-13 | GCA 030122865.1 | 4.1 | 4214 |
| *Bacillus subtilis* SX01705 | GCA 002216085.1 | 4.1 | 4318 |
| *Bacillus subtilis* CW14 | GCA 002163815.1 | 4.3 | 4329 |

Table S2 The genes involved in stress resistance in *B*. *subtilis* YZ01 genome.

| Location | Gene name | Length (bp) |
| --- | --- | --- |
| catalase |  |  |
| ctg1: 384567: 386210: +  ctg2: 010144: 011595: - | *katA* | 1644  1452 |
| ctg1: 425460: 427520: - | *katE* | 2061 |
| Mn-containing catalase |  |  |
| ctg2: 364708: 365544: +  ctg4: 000771: 001625: +  ctg4: 145315: 146136: + | *ydbD* | 837  855  822 |
| ctg7: 189008: 189577: + | / | 570 |
| RNA polymerase σ factor |  |  |
| ctg1: 389881: 390417: - | *sigY* | 537 |
| ctg2: 083315: 083806: -  ctg3: 060167: 060703: - | *sigM* | 492  537 |
| ctg2: 458698: 459453: + | *sigI* | 756 |
| ctg3: 089076: 089576: +  ctg7: 082757: 083290: + | *sigV* | 501  534 |
| ctg4: 117173: 117961: - | *sigB* | 789 |
| ctg4: 440970: 441533: - | *sigW* | 564 |
| ctg5: 190816: 191394: + | *sigO* | 579 |
| ctg6: 224753: 225517: - | *sigD* | 765 |
| ctg6: 335583: 336365: - | *sigG* | 783 |
| ctg6: 336505: 337224: - | *sigE* | 720 |
| ctg8: 016641: 017225: + | *sigX* | 585 |
| ctg9: 010706: 011473: - | *sigF* | 768 |
| ctg9: 166667: 167782: - | *sigA* | 1116 |
| ctg9: 219554: 220282: + | *sigK* | 729 |
| ctg10: 015503: 016159: + | *sigH* | 657 |
| spore coat protein |  |  |
| ctg1: 116152: 117243: -  ctg2: 201743: 202666: - | *cotH* | 1092  924 |
| ctg1: 117386: 118138: + | *cotG* | 753 |
| ctg1: 312389: 312916: + | *gerQ* | 528 |
| ctg1: 601710: 602189: + | *cotF* | 480 |
| ctg2: 299469: 299918: - | *cotZ* | 450 |
| ctg2: 300046: 300534: - | *cotY* | 489 |
| ctg2: 300685: 301200: - | *cotX* | 516 |
| ctg2: 301294: 301617: - | *cotW* | 324 |
| ctg2: 301658: 302044: - | *cotV* | 387 |
| ctg2: 326902: 327150: - | *cotT* | 249 |
| ctg6: 080056: 080430: + | *cotM* | 375 |
| ctg6: 166323: 166868: - | *cotE* | 546 |
| ctg7: 072013: 073554: - | *cotA* | 1542 |
| serine/threonine-protein kinase |  |  |
| ctg4: 119816: 120217: - | *rsbT* | 402 |
| RsbT co-antagonist protein |  |  |
| ctg2: 434001: 434834: + | *rsbRB* | 834 |
| ctg4: 120221: 120586: - | *rsbS* | 366 |
| ctg4: 120591: 121412: - | *rsbRA* | 822 |
| ctg8: 176534: 177382: - | *rsbRC* | 849 |
| ctg9: 130462: 131301: - | *rsbRD* | 840 |
| anti-sigma B factor antagonist |  |  |
| ctg4: 118406: 118735: - | *rsbV* | 330 |
| serine-protein kinase |  |  |
| ctg4: 117927: 118409: - | *rsbW* | 483 |
| phosphoserine phosphatase |  |  |
| ctg2: 165002: 165583: - | *pspA* | 582 |
| ctg2: 429450: 430076: + | *pspB* | 627 |
| ctg4: 116574: 117173: - | *rsbX* | 600 |
| ctg4: 118797: 119804: - | *rsbU* | 1008 |
| ctg5: 099874: 101085: - | *rsbP* | 1212 |
| cold shock protein |  |  |
| ctg2: 034630: 034833: - | *cspB* | 204 |
| ctg4: 094242: 094445: - | *cspC* | 204 |
| ctg8: 123803: 124003: - | *cspD* | 201 |
| ferredoxin |  |  |
| ctg8: 021875: 022123: - | *fer* | 249 |
| thioredoxin |  |  |
| ctg3: 276525: 276839: - | *trxA* | 315 |
| Cu/Zn-SOD |  |  |
| ctg8: 182810: 183397: + | *yojM* | 588 |
| Mn-SOD |  |  |
| ctg9: 152939: 153547: - | *sodA* | 609 |
| phytoene synthase |  |  |
| ctg2: 209756: 210562: + | *yisP* | 807 |
| antitoxin |  |  |
| ctg3: 188249: 188707: + | *yobK* | 459 |
| ctg4: 121885: 122166: - | *endoAI* | 282 |
| ctg7: 176437: 176865: -  ctg7: 176888: 177343: -  ctg7: 177442: 177912: -  ctg7: 177958: 178401: -  ctg7: 178418: 178825: - | *yezG* | 429  456  471  444  408 |
| K^+^/H^+^ antiporter |  |  |
| ctg2: 115163: 116380: - | *khtU* | 1218 |
| ctg2: 116388: 116885: -  ctg3: 145616: 146113: - | *khtT* | 498 |
| ctg2: 116943: 117281: - | *khtS* | 339 |
| Na^+^/H^+^ antiporter |  |  |
| ctg2: 098843: 100204: -  ctg6: 132549: 133838: - | *nhaC* | 1362  1290 |
| ctg5: 351793: 352167: - | *mrpG* | 375 |
| ctg5: 352151: 352435: - | *mrpF* | 285 |
| ctg5: 352435: 352911: - | *mrpE* | 477 |
| ctg5: 352917: 354407: - | *mrpD* | 1491 |
| ctg5: 354391: 354732: - | *mrpC* | 342 |
| ctg5: 354732: 355163: - | *mrpB* | 432 |
| ctg5: 355156: 357561: - | *mrpA* | 2406 |
| Ca^2+^/H^+^ antiporter |  |  |
| ctg7: 292280: 293335: + | *chaA* | 1056 |
